# Numbers and Characteristics of Cats Admitted to Royal Society for the Prevention of Cruelty to Animals (RSPCA) Shelters in Australia and Reasons for Surrender

**DOI:** 10.3390/ani6030023

**Published:** 2016-03-16

**Authors:** Corinne Alberthsen, Jacquie Rand, John Morton, Pauleen Bennett, Mandy Paterson, Dianne Vankan

**Affiliations:** 1 School of Veterinary Science, University of Queensland, Gatton 4343, Australia; j.rand@uq.edu.au; 2 Jemora Pty Ltd, PO Box 2277, Geelong 3220, Australia; john.morton@optusnet.com.au; 3 School of Psychological Science, La Trobe University, Bendigo 3550, Australia; Pauleen.Bennett@latrobe.edu.au; 4 Royal Society for the Prevention of Cruelty to Animals (RSPCA), Wacol Animal Care Campus, Wacol 4076, Australia; mpaterson@rspcaqld.org.au (M.P.); dianne.stephens@outlook.com (D.V.)

**Keywords:** cat, animal shelter, surrender, sterilization, excess pets, relinquishment

## Abstract

**Simple Summary:**

National Royal Society for the Prevention of Cruelty to Animals (RSPCA) shelter admission data were utilized to examine cats presented to Australian animal shelters and reasons for surrender. This study reports the most commonly cited reasons for an owner to surrender and found lower than expected sterilized cats.

**Abstract:**

Despite high numbers of cats admitted to animal shelters annually, there is surprisingly little information available about the characteristics of these cats. In this study, we examined 195,387 admissions to 33 Australian RSPCA shelters and six friends of the RSPCA groups from July 2006 to June 2010. The aims of this study were to describe the numbers and characteristics of cats entering Australian RSPCA shelters, and to describe reasons for cat surrender. Data collected included shelter, state, admission source, age, gender, date of arrival, color, breed, reproductive status (sterilized or not prior to admission), feral status and surrender reason (if applicable). Most admissions were presented by members of the general public, as either stray animals or owner-surrenders, and more kittens were admitted than adults. Owner-related reasons were most commonly given for surrendering a cat to a shelter. The most frequently cited owner-related reason was accommodation (*i.e.*, cats were not allowed). Importantly, although the percentage of admissions where the cat was previously sterilized (36%) was the highest of any shelter study reported to date, this was still lower than expected, particularly among owner-surrendered cats (47%). The percentage of admissions where the cat was previously sterilized was low even in jurisdictions that require mandatory sterilization.

## 1. Introduction

Although it can be argued that euthanasia is an essential humane option for very ill, injured, or feral cats unable to be legally released or rehabilitated as a domestic pet, many cats that are euthanized in shelters are socialised, and healthy (or treatable), and are therefore suitable for rehoming [1,2].

The owned domestic cat population in Australia was predicted to be in decline [3] and 90% or more of the owned population is reported to be sterilized [4,5,6]. Hence, a corresponding reduction in annual numbers of cats admitted to shelters and, therefore, a reduction in the numbers of cats euthanized annually might be expected. This prediction is not supported by previously available data, as Australian Royal Society for the Protection and Care of Animals (RSPCA) shelters have reported no substantial change in numbers admitted annually, and euthanasia has remained high [7]. It seems that, despite attempts to control domestic cat populations, there has been limited success in reducing annual numbers of cat admissions and euthanasia in Australia [1,7,8].

A possible explanation for these data is that previous Australian research indicates the majority of cats entering shelters in the states of Queensland and Victoria are recorded as strays [1,2]. If the majority of cats admitted to shelters are truly stray, traditional programs to reduce the excess cat population that focus on reproductive control of owned cats (early age de-sexing, low cost de-sexing clinics and vouchers, responsible ownership promotion, *etc.*) will have limited success in reducing annual numbers of cat admissions [2]. Knowing where to effectively focus management programs for cats is important because reducing the numbers of cats entering shelters annually will potentially reduce the numbers of cats euthanized each year [9]. In the U.S., a decrease in annual numbers of dogs euthanized in Californian animal care and control agencies between 1970 and 1995 was reportedly achieved almost entirely by reducing annual shelter intake numbers [9]. A thorough understanding of the complex problem of excess cats is required to determine where resources should be directed for maximum benefit [10]. Detailed information on admission patterns to shelters on a national level, however, has not been previously reported in Australia. Identifying the sources and modes of cat admissions to shelters, the reasons for relinquishment in Australian shelters and admission patterns on a national level will help stakeholders create effective strategies to reduce cat entry to shelters. This information could also inform design of such strategies in other areas such as North America and the United Kingdom, and might assist in reducing the excess pet problem on an international scale. 

Australia is a socially, culturally, and geographically diverse continent [11] that covers approximately 7.69 million km^2^ and supports a human population of approximately 22.7 million [12]. The legislative responsibility for the management of companion animals (primarily cats and dogs) in Australia lies with local government [13] within the country’s six states (Queensland, New South Wales, Victoria, Western Australia, South Australia, Tasmania) and two territories (The Australian Capital Territory and The Northern Territory) and as such, variations in companion animal management are common. With relevance to the current study, the only Australian state or territory with mandatory sterilization of cats is the Australian Capital Territory (all dogs and cats must be sterilized by the age of 6 months unless a specific breeding permit is held by the identified owner) [14].

The aims of this study were to describe the numbers and characteristics of cats entering Australian RSPCA shelters, and to describe reasons for cat surrender.

## 2. Experimental Section

### 2.1. Study Overview

A retrospective study was conducted using data collected between 1 July 2006 and 30 June 2010 from cats entering RSPCA shelters in Australia that used a standard animal management database (ShelterMate^©^, RSPCA Qld, Wacol, Australia). Data for all cats entering the shelters were collected from the database, and numbers and characteristics of cats and reasons for surrender described. The methodology for this study was based on that used by Corinne Alberthsen and Jacquie Rand [2].

### 2.2. Shelter and Admission Selection

Of 46 RSPCA shelters (including 6 friends of shelters groups) operating in Australia, all but seven small shelters in regional Victoria were using the standard animal management database (ShelterMate^©^) during the study period; these seven Victorian shelters were not included in the study. All other RSPCA shelters (*n* = 33), and six friends of the RSPCA groups (these are generally made up of volunteers that assist the RSPCA with fostering, rehoming, support and fundraising) were included.

Of the shelters included in the study, only the Malaga shelter in Western Australia did not accept all cats presented. This shelter operated as a no-kill shelter and only accepted owner-surrendered and injured cats (*i.e.*, no feral or healthy stray cats were taken in [15]). A fee was charged at all shelters for the admission of owned adult cats, owned kittens, or litters of kittens. However, no animals were refused admission to any shelter if the fee was unable to be paid (RSPCA 2008). Fees charged varied from $15 to $60, depending on the state and shelter and, at some shelters, fees varied between adult cats, kittens, and multiple entries (surrenders consisting of more than 1 adult cat or a queen and litter).

A cat admission was defined as an adult cat or kitten arriving alive and being admitted to any study shelter on or between 1 July 2006 and 30 June 2010; all admissions were entered as individuals regardless of being presented as a multiple entry; and all admissions other than cats admitted as private boarding animals and as the exceptions described below were enrolled. Based on RSPCA cat identification numbers, some cats were admitted more than once during the study period; only first admissions for these cats were included in data analyses. In Victoria, no cats were recorded as being admitted to Victorian RSPCA shelters for the months of July, August, September, and October, 2006, but 5000 cats were recorded as being admitted in December 2006. This was due to the migration of Victorian RSPCA data onto the ShelterMate^©^ system during this year. In addition, shelters in South Australia had only been utilising ShelterMate^©^ since 2007. As a result, admissions in the first study year (July 2006–June 2007) to shelters in Victoria and South Australia were excluded from analyses of numbers of admissions by month and between years, but year 1 Victorian data were included in all other analyses.

### 2.3. Data Collection

Data had been entered onto the ShelterMate^©^ database by RSPCA staff at the time of each cat’s admission, using a combination of drop down options and free text fields. The data entry software was consistent across states throughout the duration of the study. Data were exported from ShelterMate^©^ and imported into a spreadsheet (Microsoft Excel) for manipulation. Data collected included cat identity code (as allocated by RSPCA staff), date of admission, state, shelter, age at admission (estimated by RSPCA general and veterinary staff), gender (male or female), breed, coat colour, reproductive status at admission (sterilized or entire), feral status at admission (feral or not), and the mode of admission to shelter (owner-surrender, stray, municipal council, *etc.* as defined below). If the mode of admissions was “owner-surrender”, RSPCA staff prompted for and recorded a primary reason for relinquishment.

### 2.4. Definitions

All cats were classified into one of 10 admission source categories and then, for descriptive purposes, were assigned to one of three main groups (general public admission, authorized personnel admissions, other admissions: see Table 1 for definitions).

Of cats admitted as owner-surrenders, surrender reasons (if provided) were recorded using a drop down menu for the most common reasons provided, with the option of using free text if the reason was unusual. These reasons were later grouped for comparison into mutually exclusive general categories [8]: owner-related, behaviour, medical, age, humane, legal, and other.

For the purposes of this study, the terms “cat” or “cats” were used to imply all cats collectively, regardless of age. Cat age at admission was either provided by owner or estimated by shelter staff and categorised as kitten or adult but, unfortunately, definitions of “kitten” and “adult” varied by the state or territory. To manage this situation, for some descriptive comparisons and analyses by age category (adult cat or kitten), shelters with the same definitions for age categories were grouped together. Age-group 1 shelters (Queensland) defined kittens as those cats estimated to be less than 3 months of age, age-group 2 shelters (Victoria) defined kittens as those estimated to be less than 4 months of age, age-group 3 shelters (Northern Territory, the Australian Capital Territory, South Australia, and Tasmania) defined kittens as those estimated to be less than 6 months of age, and age-group 4 shelters (New South Wales and Western Australia) defined kittens as those estimated to be less than 12 months of age.

Cats were classified by breed. Each was classified based on appearance and owner information as being one of pure-breed (all cats recorded as pure breed were grouped together) or, for non-pure breeds, domestic short haired, domestic medium haired, domestic long haired (all based on coat hair length). All cats that were not assigned a specific breed or type were grouped into a category called other.

Each cat was classified by coat colour into tortoiseshell, tabby, seal point, or one of 13 categories differentiated based on a solid colour (black, white, blue, brown, orange), solid colour and white (*i.e.*, black and white, blue and white, *etc.*), or solid colour with a point (*i.e.*, black with colour point, blue with colour point, *etc.*). Categories were mutually exclusive and cats could only fit the description of one category.

Year of admission was defined using Australian financial years, with each year beginning on 1 July and ending on the 30 June: year 1 (July 2006–June 2007), year 2 (July 2007–June 2008), year 3 (July 2008–June 2009) and year 4 (July 2009–June 2010).

Feral status at entry had been recorded by RSPCA staff members using a drop down menu with “yes” or “no” options. Approximately 87% of all cat admissions had been categorised in this way, with only 13% having no data recorded in this field. Unstructured interviews with RSPCA staff from 14 shelters around Australia were conducted by the researcher, regarding how each shelter defined a feral cat. Although criteria varied between shelters, cat behaviour was the major determinant for classifying cats as feral; cats that were extremely aggressive and unable to be handled by RSPCA staff were typically classified in this way. This meant that any cat admitted to an Australian RSPCA shelter could be categorised as feral if the behaviour of the cat was deemed to be extreme, regardless of admission mode (*i.e.*, an owner-surrendered cat could potentially be categorised as feral). Cats categorized as feral did not have results of behavioral assessment for sociability recorded.

Cats were retrospectively categorised as having been sterilized prior to admission or not, based on a modified version of the methodology described in Corinne Alberthsen and Jacquie Rand [2]. This method was devised as the data field ’sterilized’ did not distinguish between cats that had been sterilized prior to admission and those admitted to shelters and then sterilized. Sterilization status recorded by shelter staff included information provided by the owner, examination for testis, tattoos or scars, or at the time of surgery. Under the modified method, cats recorded as “yes” for sterilized and without a recorded sterilization date were assumed to have been sterilized prior to admission. Cats with “no” for sterilized, or “yes” with a sterilization date on or after admission date, were assumed to have been sexually entire on admission. No cat had “yes” for sterilized and a sterilization date that was prior to their admission date. The pre-admission sterilization status of all other cats was unable to be reliably determined.

### 2.5. Statistical Analyses

Associations between admission source and each of the binary dependent variables, sterilized prior to admission, feral status and gender, were assessed using univariable logistic regression with the -xtmelogit- command in Stata (version 11.2; 2009) [16], with the individual cat used as the unit of analysis, and with clustering of outcome variables by shelter accounted for by fitting shelter as a random effect in each model. For these analyses, admission source categories were stray, owner-surrender, council (the three most common categories) and other (those cats not admitted by one of these three sources). Overall significance of admission source was assessed using likelihood ratio tests. Distributions of cats by admission source (stray, owner-surrendered, euthanasia request, returns, council, ambulance, humane officer, bequest, foster offspring, shelter offspring, transfer in, and no recorded admission) were compared between kitten and adult admissions using likelihood ratio chi-square tests [17]. Two-tailed exact binomial goodness of fit tests [17] were used to assess whether the ratios of males to females entering shelters differed from 50:50. Comparisons by sex were performed separately for all cats, adult cats, and kittens; only those cats with a recorded sex were included in these analyses. The unit of analysis was the individual first admission for all of these analyses. The comparisons of admission sources between kittens and adult cats and assessments of ratios of males to females should be considered as approximate only, as these analyses did not account for any clustering of admissions within cat and cat within shelter.

Numbers of admissions per shelter by calendar month were assessed separately for each of all admissions, adult cat admissions and kitten admissions using linear regression with shelter fitted as a random effect, using the -xtreg- command in Stata, with maximum likelihood estimation. The unit of analysis was the shelter-month; data from one month from one shelter constituted a shelter-month. December was used as the reference group in this analysis as this month had the highest number of cats admitted for the study period overall. Numbers admitted were log (base e)-transformed before analysis and regression coefficients were exponentiated to provide estimates of patterns in numbers of admissions per shelter by month expressed as ratios of geometric means for each calendar month relative to December. For all three models, to account for any long term trends in numbers of admissions per shelter, study month number was fitted as a covariate where July 2006 was study month number 1 and June 2010 was study month number 48; linear and quadratic terms were fitted. Homoscedasticity and normality of residuals were assessed for all linear regression models by visual examination of scatterplots of residual *versus* fitted values and histograms of residuals. Residuals were assessed both without and in combination with the random error component due to the random effect. Residuals were homoscedastic and approximately normally-distributed for all three models.

## 3. Results and Discussion

A total of 191,512 individual cats contributed 195,387 admissions to the 33 Australian RSPCA shelters and six friends of the RSPCA groups between 1 July 2006 and 30 June 2010. The majority of cats (191,512) were admitted to a shelter only once. However 3620 (2%) cats were admitted twice, 230 three times, 19 cats four times, three cats five times, one cat six times and one cat had a total of seven admissions. Multiple admissions for the same cat were detected based on microchip data; all cats rehomed by the shelter after an initial admission were microchipped before leaving the shelter and only cats that were microchipped and reclaimed were able to be identified as re-admissions. Some cats may have been admitted more than once; however, if they were reclaimed and not microchipped this would not have been recorded.

The total number of admissions to RSPCA shelters each year was relatively consistent throughout years 2–4 (2007/2008 to 2009/2010) ranging from 52,976 to 52,617 admissions. Of all admissions, 47% were recorded as adult cats and 53% as kittens (Table 2). The proportions of cat admissions that were kittens differed between shelters grouped by definition of kitten, but the greatest proportion of admissions that were kittens did not occur in the shelters with the oldest age definition of kitten, indicating that factors other than estimated ages also affect the proportion of admissions recorded as being kittens. In age-group 1 shelters (kittens were defined as those ≤3 months) and age-group 4 shelters (kittens = ≤12 months), more admissions were recorded as kittens (53% and 64%, respectively, in age-group 3 shelters (kittens = ≤6 months) approximately equal proportions of admissions were adult cats and kittens, and in age-group 2 shelters (kittens = ≤4 months) only 40% of admissions were kittens.

Of all admissions with a recorded gender (87% of all admissions), 52% were female and 48% male. This was significantly different from the expected 50:50 gender proportion (*p* < 0.001). This overrepresentation of female cats was evident for kittens and adult cats, and within each age-group.

Breed was recorded for all admissions and domestic short haired cats were the most common breed category (79%). This was true for adult cats and kittens. Very few adults (8%) or kittens (2%) were recorded as pure breed.

Coat colour was recorded for 99% of admissions, with the most common coat colours being tabby (34%), followed by black (26%) and tortoiseshell (11%). This order of predominance of coat colour was consistent over age categories (adult cats and kittens, and within each age-group).

### 3.1. Admission Source

Admission source was recorded for every admission. The proportions of admissions for each mode of admission were similar in each year of the study period. The majority (81%) of all admissions were from the general public. Of these, 58% were strays and 39% owner-surrendered (Table 2). Authorised personnel presented 18% of all admissions, of which most (71%) were from municipal councils. This was associated with a formal agreement with the RSPCA in which cats were transferred immediately, or after the state-legislated minimal holding period (Table 2). Very few (1%) were transferred from other organisations.

Most (84%) kitten admissions were from the general public, of which 62% were stray and 37% were owner-surrendered. Similarly, for adult cat admissions, most (75%) were from the general public; however, the proportions of these that were strays (53%) and owner-surrendered (43%) were more similar than for kittens (Table 2). A higher percentage of adult cat admissions (24%) were presented by authorised personnel compared to kittens (14%) (Table 2).

Gender ratios differed between admission sources. Proportions of admissions that were female exceeded 50% for cats admitted as strays (52%), owner-surrender (55%), by a humane officer (53%), euthanasia request (57%), or as a return (52%). In contrast, the proportion of admissions that were male exceeded 50% for cats admitted as ambulance admissions (54%), and for cats born in shelter (52%). Of cats transferred in or admitted by a municipal council, 50% were male and 50% female. There was little difference between the proportions of kittens that were male and female amongst those admitted as strays or owner-surrenders within every age-group.

### 3.2. Feral

Ten percent of 169,222 admissions were classed as feral (87% of 195,387 admissions had feral status recorded as yes or no). (Table 3). This was consistent throughout the study period. When separated by age, a higher percentage of adult cat admissions (12%) than kittens (8%) were categorised as feral (Table 3). Of those admissions where the cat was classified as feral, the largest proportion were strays (61%) followed by council admissions (19%) and owner-surrenders (11%). However, when examining the percentage of admissions where the cat was feral by admission category, ambulance admissions had the highest percentage that were feral (23%). Council admissions had the next highest percentage (15%), followed by strays (13%) (Table 3). The odds of council cat admissions being feral were 1.5 (95% CI 1.4 to 1.6) times higher than for stray admissions (*p* < 0.001). As might be expected, owner-surrenders had much lower odds of being categorised as feral compared to stray admissions (Odds ratio (OR) 0.2, 95% CI 0.2 to 0.2; *p* < 0.001).

### 3.3. Sterilization Status

Overall, only 55% (107,856/195,387) of admissions could be categorised as having been sterilized (*i.e.*, spayed or neutered) or entire prior to admission (Table 4). Of the 107,856 categorised admissions, 36% were categorised as having been sterilized prior to admission (38% and 39% of females and males, respectively; Table 4). The percentage of admissions where the cat had been sterilized prior to admission decreased during the study period, from 40% of all admissions in 07/08 to 32% in 09/10. A higher percentage of adult cat admissions were categorised as sterilized prior to admission (50%) than kitten admissions (22%) (Table 4 and Table 5). As expected, as the age definition of kitten admissions increased in each age-group, so did the percentage that were sterilized prior to admission (age-group 1 (kittens ≤3 months) = 7%, age-group 2 (kittens ≤ 4 months) = 26%, age-group 3 (kittens ≤ 6 months) = 30%, and age-group 4 (kittens ≤ 12 months) = 38%).

Of admissions as owner-surrenders, 47% (64% of adult cats and 27% of kittens) had been sterilized prior to admission (Table 5). In comparison, 39% of council admissions, and 24% of stray admissions were recorded as sterilized prior to admission. Overall, owner-surrendered admissions had 2.6 (95% CI 2.5 to 2.7; *p* < 0.001) times higher odds of being sterilized prior to admission, relative to stray admissions. Council admissions had only modestly higher odds of being sterilized prior to admission relative to stray admissions (OR 1.6, 95% CI 1.5 to 1.7; *p* < 0.001). Of stray adult cat admissions, 35% were categorised as sterilized prior to admission, indicating that a substantial proportion of cats admitted as strays may have been owned previously.

### 3.4. Surrender Reasons

Of all 195,387 admissions, 32% (61,755) were owner-surrenders. Of owner-surrenders, 80% (49,393) had a reason for surrender provided. Results in this section describe percentages of those 49,393 admissions. Of those admissions, the majority were for owner-related reasons (91%). This was true for all admissions combined and within adult and kitten admissions separately (Table 6). The percentage of admissions that were for owner-related reasons was higher for kittens (95%) than adults (87%) (Table 6). Of admissions for owner-related reasons, the most frequently cited reason was for accommodation restrictions (pets not allowed) (21%) followed by “too many animals” (18%) (Table 6). Accommodation restrictions were the most common for adult cats (36%), while “own litter” was more likely for kittens (28%). This implies that owners could not find homes for kittens bred from a cat they identified as their own (Table 6). An additional 16% of kitten admissions surrendered for owner-related reasons were classified by the owner as unwanted and a further 22% were surrendered because of too many animals. Thus, of kitten admissions for owner-related reasons, approximately 66% were surrendered because the kitten(s) were in excess of the numbers of cats desired by the owner.

Interestingly, behaviour accounted for only 4% of admissions. Of all admissions surrendered for behavioural reasons, the most common were for inappropriate elimination (22%), aggressive behaviour (21%) and for being unfriendly and un-socialised (20%). A higher percentage of adult cat admissions (7%) were surrendered for behavioural reasons than kittens (1%) (Table 6). Inappropriate elimination was the most common cause of surrender for behavioural reasons in both adults and kittens, with unfriendly/un-socialised behaviour and aggression also prominent.

Other reasons for surrender were legal requirements (2% of owner-surrenders where a reason for surrender was provided), cat health (1%), cat age (1%) and humane reasons (1%) (Table 6). The most frequent health reasons were for unspecified illness (48%) or other unspecified medical reason (22%), followed by cancer (14%). Of admissions where the cat was surrendered for age-related reasons, old age was by far the most common (78%). Very few (<1%) kittens were surrendered because of health or age-related reasons.

### 3.5. Seasonal Patterns in Numbers of Admissions

**Figure 1:**
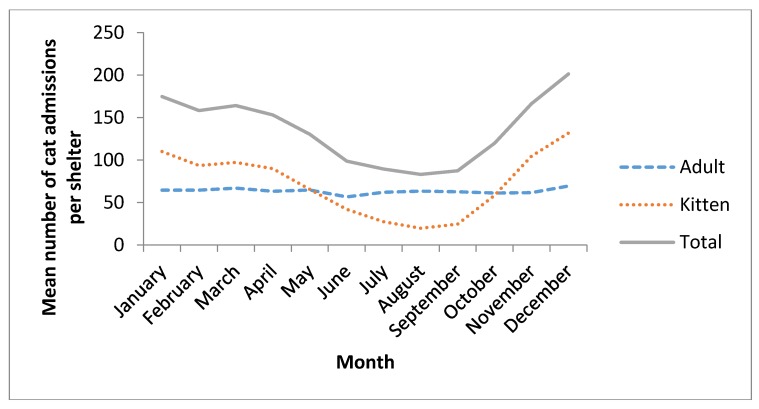
Mean numbers of cat admissions per RSPCA shelter per month between June 2006 and July 2010 by age category and calendar month of admission.

Cats were admitted every month throughout the study period; however, there was a distinct peak in numbers admitted per shelter from November through to April (late spring to autumn; Figure 1 and Table 7). For all admissions over the 4 year study period, the month with highest arithmetic mean number of cat admissions per shelter was December (mean total number of admissions per shelter over the 4 study years: 201). Lowest mean numbers were in the cooler months of late winter/early spring, with August (mean number of admissions per shelter 83) being the month with least admissions (Figure 1 and Table 7).

**Figure 2:**
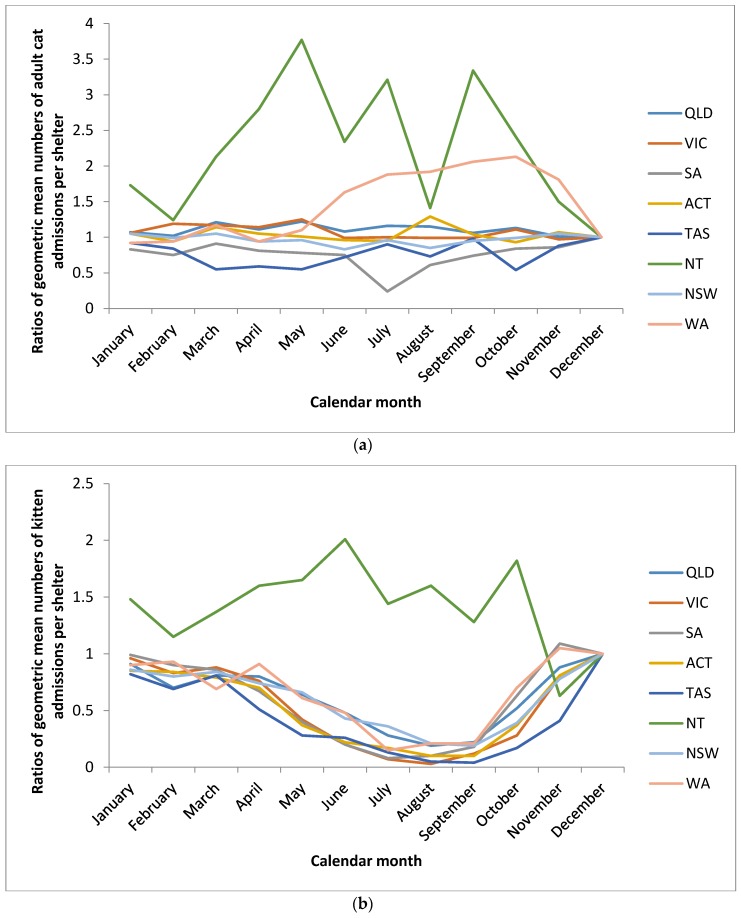
Ratios of geometric mean numbers of cat admissions per RSPCA shelter per month for January to November relative to December by state/territory. (**a**) Ratios of geometric mean numbers of Adult Cat admissions per RSPCA shelter per month for January to November relative to December by state/territory; (**b**) Ratios of geometric mean numbers of Kitten admissions per RSPCA shelter per month for January to November relative to December by state/territory. (Queensland (QLD), Victoria (VIC), South Australia (SA), Australian Capital Territory (ACT), Tasmania (TAS), Northern Territory (NT), New South Wales (NSW), and Western Australia (WA)). * For example, in Northern Territory (NT), the geometric mean number of adult cat admissions per shelter in January was 1.7 times higher than that in December.

For adult cats, there was minimal difference between the arithmetic mean numbers admitted per shelter in December (70) and September, the month with the lowest mean number of adult cat admissions per shelter (63) (Figure 1). Numbers of adult cat admissions were relatively constant by months in all states and territories except Western Australia and the Northern Territory (Figure 2a). Western Australia demonstrated a prominent increase in adult admissions from June to November. In the Northern Territory, the mean number of cat admissions per shelter was markedly higher in all months compared to December.

For kitten admissions, the highest monthly arithmetic mean number of kitten admissions per shelter was in December (132) and the lowest mean was in August (20) (Figure 1). In contrast to adult admissions, mean kitten numbers admitted differed markedly by month (*p* < 0.001), and exhibited a distinct and markedly similar seasonal pattern, regardless of state/territory (despite definitions of kitten varying by state), with the exception of the Northern Territory (Figure 2b). Most kittens were admitted between October through to April (late spring, summer and early autumn) with a distinct dip in admissions from May to September (late autumn, winter and early spring) (Figure 2a,b). Similar to adult admissions, the mean number of kitten admissions per shelter in the Northern Territory was much higher in all other months compared to December and did not display a seasonal dip in mean number of admissions per shelter (Figure 2b).

## 4. Conclusions

One of the most important findings of this study was that most admissions (80%) were presented to shelters by members of the general public as either a stray or as an owner-surrendered cat. This finding indicates that strategies to reduce admissions to shelters need to target the general public. However, they need to be optimised for the two different cat populations: stray and owned, because strategies aimed at owned cats may not be effective for stray cats.

General public cat admissions in our study (80% of all admissions) were a higher proportion than reported from two shelters in South Australia—69% of 13,300 cats—and from a large feline-only shelter in Melbourne, Victoria—55% of 15,206 cats (37% public stray, 18% owner-surrendered) [1,8]. This may reflect the types of shelters studied, as admissions have been found in the U.S. to be influenced by the type of shelter operation. For example, some studies report mostly general public admissions (both stray and owned), while municipal pounds have predominately authorised personnel admissions [18,19]. RSPCA shelters in Australia can perform both functions; some shelters also run a municipal pound under contract from local municipal councils.

Stray cats presented by members of the general public accounted for the greatest single source of admissions (47% of all admissions; 52% of kitten admissions and 40% of adult cat admissions). Few cats (10%) were classified as feral, and therefore presumably most stray cats had contact with humans on a regular basis. This information is important because if stray cats are truly un-owned, then strategies aimed at owners and owned cats will have little effect on the largest portion of shelter admissions [2].

Semi-owned cats have been described in the literature as cats that receive some care, but the carer does not take responsibility for the cat [5]. Australian research on community attitudes and beliefs towards cats found that 22% of phone survey respondents admitted to feeding a cat that they did not own, almost as many as those who claimed to own a cat (33%) [5]. In an Australian online survey involving an educated and high socioeconomic population, 10% of respondents were classed as semi-owners because they did not perceive themselves as the owner of the cat but had interacted with it for at least a month and fed the cat frequently or always [20]. A study that surveyed 177 people in Brooklyn, New York, found that 22% of respondents fed a free-roaming cat [21], and a survey of current U.S. pet owners found 16% care for stray cats, with 97% feeding them (American Pet Product Association). In U.S.A. it is estimated that 44% of the cat population is semi-owned [22], and based on the Australian data, it could represent two thirds the size of the owned cat population [5]. The proposal that a semi-owned sub-population of cats in the community is at least partly responsible for a substantial proportion of shelter admissions is supported by the finding that 59% of people surrendering a stray cat to the RSPCA in Australia provided some care for the cat, and 33% had been associated with the cat for more than a month [23,24]. Surrenderers of both stray (54%) and owned cats (24%) also fed one or more other unowned cats in the previous 5 years that were not surrendered. People surrendering stray cats more often did so because they are concerned for the cat (72%) or thought it was better off in a shelter (59%), whereas people surrendering owned cats did so largely for human-related reasons including accommodation-related, personal or financial reasons [23]. Semi-owned cats are less likely to be sterilized and more likely to have had a litter of kittens than owned cats, with 30% of semi-owned and 7% of owned cats having had kittens [20]. Understanding semi-owned cat populations and their interactions with humans in the community is essential when formulating and implementing strategies to reduce shelter admissions.

Although most cats classified as feral were stray, a portion of feral cats were admitted as owner-surrenders (6%) for owner-related reasons rather than behaviour (as would be expected if the cat was truly feral). This indicates that classification of feral status is possibly inaccurate, as previously suggested by Corinne Alberthsen and Jacquie Rand [2]. The classification of cats as feral in the RSPCA shelters included in this study was based on a subjective behavioural assessment and criteria for classifying cats as a feral cat probably differ between shelters and even between assessors within shelters, depending on the experience, training and opinions of the person performing the assessment [2,25,26]. Cat behaviour and disposition on admission to a shelter vary considerably and at the time that the current study was conducted, no validated measure for distinguishing truly un-socialised and feral cats from frightened pet cats was available [26,27]. Methods for this have been developed since [28]. Further, in one study, owner-surrendered cats displayed higher behavioural measures of stress than stray cats on admission to shelters [27]. A nationwide U.S. study that examined the methods used by welfare organisations to evaluate and categorise cat admissions found that the shorter the holding period, the higher the risk of misclassification. Development of a standardised and validated procedure for identifying truly feral cats would help to minimize such misclassification errors and consequent euthanasia [26]. This is particularly pertinent, as cats that are categorised as feral are legally (in most localities) exempt from the usual holding periods and can be euthanized immediately.

After strays, owner-surrender was the second most common mode of cat admission to shelters, comprising 32% of all admissions. Surrender reasons given for cats relinquished to Australian RSPCA shelters in this study indicate that most relinquishments are due to owner-related reasons. Only a few cats were surrendered for reasons related to the animal, such as behaviour, health, and age. The most commonly cited owner-related reason for the surrender of a cat to an RSPCA shelter was for accommodation-related reasons. This is consistent with previous Australian research [1]. In the U.S., human-related factors, particularly moving (*i.e.*, changing accommodation), are also reported as prominent reasons for the relinquishment of pet cats [29,30,31]. In two studies, moving was ranked as the most common or third most common reason [29,30]. As in most developed countries, the population of Australia is moving away from rural communities into urban areas and cities, with unit and apartment living becoming more popular. Many body-corporate by-laws in Australia do not allow pets to be kept in rental properties. This may be having a substantial impact on people’s ability to maintain care for their cat, and could be a causal factor behind the reported decline in cat ownership in Australia [32,33]. A nationwide U.S. study that examined the availability of “pet-friendly” rental accommodation reported that 52% of landlords surveyed allowed cats, although most imposed some sort of restriction, limitation, or additional “pet deposit” charge [34]. It was found that, overall, tenants with pets in “pet-friendly” rental accommodation stayed significantly longer than those occupying rental accommodation that prohibited pets. These results indicate that “pet-friendly” accommodation may be more profitable in the long term than accommodation that prohibits pets. If the availability of “pet-friendly” rental accommodation were increased, the surrender of many cats might be prevented, and additional homes for other unwanted and excess cats would potentially become available.

Surprisingly, behaviour accounted for very few cat surrenders in our study (4% of admissions). Other Australian research investigating 27,511 cat admissions to the South Australian RSPCA and Animal Welfare League (AWL) shelters over a two year period also reported that only 3.7% of surrenders were for behavioural reasons [8]. An identical result (3.7% of cats surrendered for behavioural reasons) was also reported by Marston and Bennett [1] in an analysis of 15,206 cat admissions to a Melbourne cat shelter in Victoria. However, unlike our study and others conducted in Australia, cat behaviour accounts for a large portion of relinquishments in the U.S. [29,30,35,36,37,38]. For example, in a study involving owners of 1,409 cats surrendered to 12 U.S. shelters, 33.2% of cats were relinquished for behavioural issues [30]. Another study investigating pet surrenders to the Ohio Humane Society of the United States, reported that 14% of 3,263 cats were relinquished due to unfavourable behaviour [29]. These differences between studies may be attributable to the limited recordable options for surrender reasons available in our study. In a study involving interviews with people surrendering pets (48% were cats) to a private shelter in Boston, Massachusetts, it was established that reasons associated with the decision to surrender a pet were complex. Often there are several reasons or more contributing to the final decision for an owner to surrender a cat, despite only one, simple reason being supplied to shelter workers [35]. Other US studies support this suggestion: 57.2% of owners had more than one reason, and up to five separate reasons for relinquishment of pet cats to 12 U.S. animal shelters [30,36]. It has been proposed that some owners surrender cats for behavioural reasons, but report a different reason because they believe the animal will have a better chance of being re-homed [29,35,39]. Another factor which might have contributed to the low number of people reporting behavioural problems as a reason for surrender is that many people surrendering a pet experience feelings of guilt [40], and attributing the reason to something beyond their control, such as accommodation issues, might help deflect blame from the relinquisher and from the pet [31].

Interestingly, many (66%) surrendered kittens (regardless of age definition) were surrendered for simply being in excess (own litter, too many cats, unwanted). This, and the fact that more than half of all admissions were identified as a kitten, indicates that, despite attempts to promote sterilization of owned cats, over-breeding is still a causal factor behind shelter admissions. In Australia, it has been reported that greater than 93% of owned cats are sterilized [3,6,41]. Despite this, there has been no overall decrease in numbers of admissions to RSPCA shelters, and the percentage of admissions in our study where the cat was sterilized prior to admission decreased with each year throughout the study period. The overall number of admissions did not decrease over time, nor did the proportions of admissions as strays or owner-surrenders change. It is unclear from this study why the percentage of admissions where the cat was categorised as sterilized prior to admission decreased over the study period. This finding could suggest a number of possibilities: sterilization messages are not reaching owners; sterilization of owned cats may be occurring after one or more litters are produced; reports on the proportions of owned cats that are sterilized may not be representative of the owned cat population in Australia; or the sterilization data presented in this study is potentially misleading due to being incomplete. As female cats can have their first oestrus from 3.5 months of age [42,43], delaying sterilization of cats may be a serious problem for the management of the domestic cat population [9,44]. Approximately 45% of sterilized cats in Massachusetts, U.S. were reportedly sterilized after 12 months of age, and the number of kittens born to cats that were eventually sterilized was not significantly different from those born to cats that remained sexually entire [44]. Other investigations have revealed that 5% of owned male cats and 13%–20% of owned female cats were known by owners to have produced at least 1 litter before they underwent sterilization [5,9].

In the current study, cats were categorised as being sterilized prior to admission for only 47% of owner-surrendered admissions (and 64% of adult cat admissions). Similarly, Marston [8] reported that, of all admissions to three shelters in South Australia between July 2007 and June 2009, 25% of cats were reportedly sterilized and, of those cats surrendered by an identified owner, 42% were recorded as being sterilized [8]. Another Australian study that investigated 15,206 cat admissions to a single shelter in Melbourne, reported that only 12.8% of owner relinquished cats were sterilized prior to admission, although admissions of unknown sterilization status were assumed to be not sterilized in this calculation [1]. Collectively, the results from these different studies suggest that delayed sterilization may be playing an important role in maintaining the excess cat problem.

Ensuring cats are sterilized before they are able to reproduce will continue to be an important strategy in reducing the number of excess and unwanted kittens admitted to shelters in Australia and the U.S. [9,45]. Many relinquished animals have visited a veterinarian prior to relinquishment [30]. Therefore, veterinarians could play a more prominent role in reducing cat admissions to shelters. This includes participating in routine early-age spay or neutering of kittens, and assisting in and educating their clients about the importance of cat sterilization and responsible pet ownership [9]. As a minority of veterinarians in Australia are trained in early-age spay or neutering techniques, and many veterinarians are reluctant to participate in this practice, it would be appropriate for professional organisations and universities to provide education and training to rectify this deficiency [2,9].

Only 55% of all admissions in our study had a recorded sterilization status on admission, and of these, for 36% of all admissions and 50% of adult cat admissions, the cat was sterilized before admission. These figures are higher than those previously reported from the U.S. and Australia. In the U.S., the cat was sterilized before admission for only 9%–13% of admissions [19,46]. Australian studies have reported 3% [1], 13% [2], and 25% [8] of admissions where the cat was recorded as having been sterilized before admission. However, most of these U.S. and Australian studies did not specify if the percentage calculations included or excluded cats of unknown sterilization status, rendering comparisons with the present study meaningless. Shore and Girrens [19] reported that shelter staff did not always collect sterilization data, resulting in only about half of all admissions being able to be categorised, as found in the present study. Regardless of the method employed to calculate the percentage sterilized prior to admission, if data is not collected accurately then it is possible that all published spay or neutering figures are over or under-reported.

The lower than expected percentage of admission where the cat was previously sterilized in our study, even in jurisdictions that have mandatory sterilization, and the fact that more than half (53%) of all admissions were under the age of 12 months, indicate that excess breeding of cats—both owned and unowned—is a major contributor to shelter admissions in Australia. Sterilization clearly plays a significant role in excess cat management, both nationally and internationally. Indeed, mandatory spay or neutering has often been considered by welfare groups, government and councils as a possible legislative requirement, to be introduced with the intention of reducing the cat population and therefore the number of cat admissions to shelters [9]. However, based on our study, mandated sterilization has limited impact. In U.S.A., a significant decrease in cat admissions and euthanasia at local shelters occurred following introduction of government-funded sterilization initiatives [45,47]. Low socioeconomic areas are overrepresented in shelter admissions [48], and an Australian study found that an association between lower socioeconomic status and having un-owned cats on one’s property, feeding un-owned cats and surrendering multiple times [24]. Semi-owned cats in Australia are less likely to be sterilized than owned cats [5,20] and are more likely to have had kittens [20]. Therefore, the efficacy of low-cost or no cost sterilization, targeted to locations over-represented by cat and kitten intake into shelters, should be evaluated in Australia.

Our results also indicate that, while numbers of adult cat admissions remain constant throughout the year, kitten admissions vary seasonally, with a distinctive peak in kitten admissions between November and April (typically the warmer months in Australia). This was true even after accounting for the differences in the definition of kittens between states. Seasonal patterns with a peak in kitten admissions in the warmer months have been reported in other studies in the southern hemisphere [1,8,48]. This is important information to consider for sterilization campaigns and social marketing messages, which may need to be intensified in cooler months (May through August) prior to the feline breeding season.

### 4.1. Limitations of This Study

Some caution is required in extrapolating these results to other countries. However, although our study was limited to Australian RSPCA shelters, we consider the results valid for Australia generally. In some localities, council pounds and other welfare organisations’ animal shelters are also in operation, often in close proximity to shelters included in this study. But, as the RSPCA is the largest and most widespread organisation operating animal shelters in Australia, it arguably provides a very good representation of the cat population entering shelters nationally. A study of this magnitude has not previously been possible. The use of the ShelterMate^©^ database was integral in enabling the analysis of data from multiple shelters.

A further limitation of this study is the validity of some of the data presented. The age of cats admitted and the identification of cats as feral or not were estimated by multiple staff members and were somewhat arbitrary. Sterilization status is potentially difficult for staff to record for female cats if a desexing tattoo was not present or had faded over time. Without a visible tattoo, it would be impossible for staff to ascertain if the cat was sterilized or not on admission. In conjunction with these discrepancies, some fields such as sterilization status, feral, and microchipped or not were not compulsory for staff to enter when admitting cats to shelters. In addition, the RSPCA database should be modified so that sterilizations performed after admission are recorded separately from sterilization status at admission. Despite the identified problems, data presented in this study provide the most comprehensive investigation of cat admissions to Australian animal shelters to date.

### 4.2. Overall Conclusions

Most cats were presented to shelters by members of the general public, as either a stray animal or as an owner-surrender, and more kittens were admitted than adult cats. It is therefore imperative that future management strategies aimed at reducing numbers of admissions be directed at influencing the general public, not cat owners *per se*. In addition, further research is required to investigate why the general public present stray cats to shelters and intervening strategies should potentially be tailored for both different ownership statuses (stray verses owned).

Owner-related reasons were most commonly given for surrendering a cat to a shelter. The most frequently cited owner-related reason was for accommodation because pets were not allowed. This has far-reaching implications for potential management as it demonstrates that reducing numbers of admissions to shelters in Australia (and, thereby reducing numbers of cats euthanized in shelters), requires strategies that focus on changing rules and potentially legislative requirements related to the inclusion of pets in rental agreements and other housing arrangements. Of the cat-related reasons, 42% were for unfriendly, aggressive or poorly socialized behaviour and 22% were for house soiling, and the frequency of these behaviours would be exacerbated, particularly in male cats, by the relatively low desexing rates.

Importantly, this study also demonstrated that, despite reporting the highest percentage of admissions where the cat was previously sterilized in any shelter study to date, this was still lower than expected, particularly among owner-surrendered cats. The percentage of admissions where the cat was previously sterilized was low even in jurisdictions that require mandatory sterilization, and even in the ACT where a mandatory sterilization policy has been in place since June 2001. These sterilization prevalences indicate that previously reported statistics for the owned cat population may be inaccurate, and that excess breeding is a significant contributor to shelter admissions. This is supported by the finding that more than half of all admissions were cats aged under 12 months. As younger cats are less likely to be sterilized than cats older than 12 months, this delay in sterilization is plausibly making a substantial contribution to the excess cat population. Early-age sterilization may continue to be an important strategy in managing excess cat populations. However, it should be noted that legislation requiring sterilization is not necessarily useful, particularly if not implemented in a timely manner. While legislation is an important tool in the management of excess pets in the community, the results of this study provide some evidence that legislative requirements do not always result in the desired outcomes. If legislative measures are not enforced or evaluated, the effectiveness of using such management strategies can be limited. Introduction of low-cost or no cost sterilization, targeted to locations over-represented by cat and kitten intake into shelters, may be more efficacious than legislation in reducing cat admissions and euthanasia in shelters.

## Figures and Tables

**Table 1:** Admission source groups, categories and definitions for cats admitted to 39 Royal Society for the Prevention of Cruelty to Animals (RSPCA) shelters between June 2006 and July 2010.

Admission Group	Admission Type	Definition
1. General public admissions	Owner-surrender	Cats presented to the shelter by the owner or agent of the owner
	Stray	Cats presented to the shelter by a person who was neither the owner nor an agent of the owner
	Bequests	Cats willed to the RSPCA by a deceased estate
	Euthanasia requests	Cats presented to the shelter by the owner requesting that the cat be euthanized
	Returns	Cats adopted from a shelter prior to the study commencement date but returned to the shelter during the study period. For a study admission to be classified as a return, the cat had to have been previously adopted from the shelter and returned within a defined period of time post adoption (generally within a month of the initial adoption), otherwise the admission was classified as an Owner Surrender.
2. Authorized personnel admissions	Ambulance	Cats that may have been reported as injured and picked up by an RSPCA animal ambulance officer
	Council	Stray, trapped and surrendered cats collected by municipal councils and admitted to an RSPCA shelter under a pound management agreement either immediately, or after the minimum holding period (usually 3–8 days depending on the state).
	Humane officer admissions	Cats brought into the shelter by an inspector for various reasons, including legal seizures and welfare reasons.
3. Other admissions	Transferred in	Cats transferred from other shelters or organizations (cats transferred from municipal councils are not included)
	Born in shelter	Cats born while in the shelter or foster care

**Table 2:** Distributions of admissions to 39 RSPCA shelters between June 2006 and July 2010 by admission mode and age.

Admission Mode	All Cats Pooled					
	Total		Adult Cats		Kittens	
**General Public**	**156,695 (81%)**		**68,658 (75%)**		**88,037 (84%)**	
	**Overall**	**Of General Public**	**Overall**	**Of General Public**	**Overall**	**Of General Public**
Stray	91,293 (47%)	91,293 (58%)	36,690 (40%)	36,690 (53%)	54,603 (52%)	54,603 (62%)
Owner-surrender	61,755 (32%)	61,755 (40%)	29,228 (32%)	29,228 (43%)	32,527 (31%)	32,527 (37%)
Euthanasia request	1837 (1%)	1837 (1%)	1587 (2%)	1587 (2%)	250 (0%)	250 (0%)
Returns	1810 (1%)	1810 (1%)	1153 (1%)	1153 (2%)	657 (1%)	657 (1%)
**Authorized Personnel**	**35,803 (18%)**		**21,626 (24%)**		**14,177 (14%)**	
	**Overall**	**Of Authorized Personnel**	**Overall**	**Of Authorized Personnel**	**Overall**	**Of Authorized Personnel**
Council	25,408 (13%)	25,408 (71%)	14,639 (16%)	14,639 (68%)	10,769 (10%)	10,769 (76%)
Ambulance	6407 (3%)	6407 (18%)	4271 (5%)	4271 (20%)	2136 (2%)	2136 (15%)
Humane officer	3988 (2%)	3988 (11%)	2716 (3%)	2716 (13%)	1272 (1%)	1272 (9%)
**Other**	**2889 (1%)**		**701 (1%)**		**2188 (2%)**	
	**Overall**	**Of Other**	**Overall**	**Of other**	**Overall**	**Of other**
Born in shelter	1568 (1%)	1568 (54%)	52 (0%)	52 (7%)	1516 (1%)	1516 (69%)
Transfer In	1321 (1%)	1321 (46%)	649 (1%)	649 (93%)	672 (1%)	672 (31%)
**Total**	**195,387**		**90,985**		**104,402**	

**Table 3:** Numbers and percentages of admissions to 39 RSPCA shelters between June 2006 and July 2010 where the cat was categorised as feral by admission mode and age. Only the 169,222 admissions where feral status (*i.e.*, not feral or feral) was recorded are included.

Admission Mode	All Cats Pooled		Adult Cats		Kittens	
	* Number (%) Categorized as Feral	Total	Number (%) Categorized as Feral	Total	Number (%) Categorized as Feral	Total
**General Public**	12,088 (9%)	138,476	5819 (10%)	61,118	6269 (8%)	77,358
Stray	10,163 (13%)	77,106	4876 (16%)	31,138	5287 (12%)	45,968
Owner-surrender	1794 (3%)	58,023	840 (3%)	27,444	954 (3%)	30,579
Euthanasia Request	112 (7%)	1679	88 (6%)	1465	24 (11%)	214
Returns	19 (1%)	1668	15 (1%)	1071	4 (1%)	597
**Authorized Personnel**	4480 (16%)	28,320	3204 (19%)	16,973	1276 (11%)	11,347
Council	3161 (15%)	21,330	2311 (19%)	12,099	850 (9%)	9231
Ambulance	1038 (23%)	4456	645 (21%)	3071	393 (28%)	1385
Humane Officer	281 (11%)	2534	248 (14%)	1803	33 (5%)	731
**Other**	42 (2%)	2426	22 (4%)	499	20 (1%)	1927
Born in Shelter	14 (1%)	1459	1 (2%)	43	13 (1%)	1416
Transfer In	28 (3%)	967	21 (5%)	456	7 (1%)	511
**All admissions pooled**	**16,610 (10%)**	***n* = 169,222**	**9045 (12%)**	***n* = 78,590**	**7565 (8%)**	***n* = 90,632**

* For example, of the 138,476 admissions from the general public where feral status was recorded, 9% (12,088) were categorised as feral.

**Table 4:** Numbers and percentages of admissions to 39 RSPCA shelters between June 2006 and July 2010 where the cat was categorised as having been sterilized prior to admission, by age and gender. Only the 107,856 admissions where sterilization status prior to admission could be determined are included.

Gender	All Cats Pooled		Adult Cats		Kittens	
	* Number (%) Categorized as Sterilized Prior to Admission	Total	Number (%) Categorized as Sterilized Prior to Admission	Total	Number (%) Categorized as Sterilized Prior to Admission	Total
**Females**	19,589 (38%)	51,748	13,836 (50%)	27,045	5753 (24%)	24,343
**Male**	19,317 (39%)	49,935	13,370 (51%)	26,240	5947 (25%)	23,695
**Sex not recorded**	135 (0%)	6173	91 (0%)	1245	44 (0%)	5288
**Total**	39,041 (36%)	107,856	27,297 (50%)	54,530	11,744 (22%)	53,326

* For example, of the 51,748 female cat admissions where sterilization status prior to admission could be determined, 38% (19,589) were categorised as sterilized prior to admission.

**Table 5:** Numbers and percentages of admissions to 39 RSPCA shelters between June 2006 and July 2010 where the cat was categorised as having been sterilized prior to admission, by admission mode and age. Only the 107,856 admissions where sterilization status prior to admission could be determined are included.

Admission Source	All Cats Pooled		Adult Cats		Kittens	
	Number (%) Categorized as Sterilized Prior to Admission	Total	Number (%) Categorized as Sterilized Prior to Admission	Total	Number (%) Categorized as Sterilized Prior to Admission	Total
**General Public**	**31,473 (36%)**	**87,525**	**22,150 (52%)**	**42,583**	**9323 (21%)**	**44,942**
*Stray*	10,856 (24%)	45,099	6845 (35%)	19,285	4011 (16%)	25,814
*Owner-surrender*	18,954 (47%)	40,182	13,826 (64%)	21,469	5128 (27%)	18,713
*Euthanasia Request*	863 (69%)	1249	861 (77%)	1115	2 (1%)	134
*Returns*	800 (80%)	995	618 (87%)	714	182 (65%)	281
**Authorized Personnel**	**7272 (39%)**	**18,868**	**4991 (43%)**	**11,623**	**2281 (31%)**	**7245**
*Council*	5459 (39%)	13,988	3419 (42%)	8065	2040 (34%)	5923
*Ambulance*	1212 (36%)	3391	1083 (44%)	2476	129 (14%)	915
*Humane Officer*	601 (40%)	1489	489 (45%)	1082	112 (28%)	407
**Other**	**296 (20%)**	**1463**	**156 (48%)**	**324**	**140 (12%)**	**1139**
Born in Shelter	115 (14%)	821	8 (35%)	23	107 (13%)	798
Transfer In	181 (28%)	642	148 (49%)	301	33 (10%)	341
**Total**	**39,041 (36%)**	**107,856**	**27,297 (50%)**	**54,530**	**11,744 (22%)**	**53,326**

* For example, of the 87,252 admissions from the general public, 36% (31,473) were categorised as having been sterilized prior to admission.

**Table 6:** Distribution of owner-surrendered admissions to 39 RSPCA shelters between June 2006 and July 2010 by age category and surrender reason. Only the 49,393 owner-surrendered admissions where a reason for surrender was provided are included. Within each column, italicised percentages sum to 100% within each surrender reason category.

Surrender Reason	Total	Adult	Kitten
**Owner-Related**	**45,009 (91%)**	**19,598 (87%)**	**25,411 (95%)**
*Owner*	*12 (0%)*	*9 (0%)*	*3 (0%)*
*Accommodation*	*9615 (21%)*	*7111 (36%)*	*2504 (10%)*
*Too Many Animals*	*8246 (18%)*	*2611 (13%)*	*5635 (22%)*
*Own Litter*	*7618 (17%)*	*438 (2%)*	*7180 (28%)*
*Unwanted*	*6014 (13%)*	*1827 (9%)*	*4187 (16%)*
*Cannot Afford*	*5174 (11%)*	*2223 (11%)*	*2951 (12%)*
*Unable To Provide Care*	*2798 (6%)*	*1991 (10%)*	*807 (3%)*
*Abandoned Animal*	*1995 (4%)*	*846 (4%)*	*1149 (5%)*
*Allergy*	*1515 (3%)*	*1043 (5%)*	*472 (2%)*
*Relationship*	*687 (2%)*	*509 (3%)*	*178 (1%)*
*Deceased*	*561 (1%)*	*493 (3%)*	*68 (0%)*
*New Baby*	*452 (1%)*	*336 (2%)*	*116 (0%)*
*Kids No Good With Animal*	*100 (0%)*	*47 (0%)*	*53 (0%)*
*Unwanted gift*	*80 (0%)*	*22 (0%)*	*58 (0%)*
*Pregnant*	*73 (0%)*	*52 (0%)*	*21 (0%)*
*Cannot Find Alternate Home*	*26 (0%)*	*11 (0%)*	*15 (0%)*
*Too Vocal*	*16 (0%)*	*13 (0%)*	*3 (0%)*
*Impulse Buy*	*11 (0%)*	*1 (0%)*	*10 (0%)*
*Sheds too much*	*8 (0%)*	*8 (0%)*	*(0%)*
*Too big*	*4 (0%)*	*4 (0%)*	*(0%)*
*Fearful*	*3 (0%)*	*3 (0%)*	*(0%)*
*Wrong Sex*	*1 (0%)*	*(0%)*	*1 (0%)*
**Behaviour**	**1834 (4%)**	**1507 (7%)**	**327 (1%)**
*Behaviour*	*109 (6%)*	*98 (7%)*	*11 (3%)*
*Inappropriate Elimination*	*410 (22%)*	*324 (21%)*	*86 (26%)*
*Aggression*	*385 (21%)*	*333 (22%)*	*52 (16%)*
*Unfriendly/Unsocialised*	*365 (20%)*	*282 (19%)*	*83 (25%)*
*Not Good With Children*	*172 (9%)*	*146 (10%)*	*26 (8%)*
*Predation*	*131 (7%)*	*121 (8%)*	*10 (3%)*
*Destructive*	*76 (4%)*	*64 (4%)*	*12 (4%)*
*Hyperactivity*	*64 (3%)*	*31 (2%)*	*33 (10%)*
*Escapes*	*57 (3%)*	*48 (3%)*	*9 (3%)*
*Fearful*	*31 (2%)*	*29 (2%)*	*2 (1%)*
*Anxiety*	*24 (1%)*	*23 (2%)*	*1 (0%)*
*Biting*	*10 (1%)*	*8 (1%)*	*2 (1%)*
**Legal**	**1044 (2%)**	**375 (2%)**	**669 (2%)**
**Medical**	**542 (1%)**	**419 (2%)**	**123 (0%)**
*Medical*	*121 (22%)*	*97 (23%)*	*24 (20%)*
*Illness*	*260 (48%)*	*197 (47%)*	*63 (51%)*
*Cancer*	*77 (14%)*	*61 (15%)*	*16 (13%)*
*Injury*	*62 (11%)*	*44 (11%)*	*18 (15%)*
*Allergy*	*6 (1%)*	*4 (1%)*	*2 (2%)*
*Feline Immunodeficiency Virus Positive*	*4 (1%)*	*4 (1%)*	*(0%)*
*Urinary Tract Infection*	*4 (1%)*	*4 (1%)*	*(0%)*
*Hair loss*	*3 (1%)*	*3 (1%)*	*(0%)*
*Surgical problems*	*3 (1%)*	*3 (1%)*	*(0%)*
*Blind*	*2 (0%)*	*2 (0%)*	*(0%)*
**Age**	**393 (1%)**	**308 (1%)**	**85 (0%)**
*Too Old*	*308 (78%)*	*306 (99%)*	*2 (2%)*
*Too Young*	*85 (22%)*	*2 (1%)*	*83 (98%)*
**Humane**	**346 (1%)**	**239 (1%)**	**107 (0%)**
*Rescued from neglect*	*164 (47%)*	*83 (35%)*	*81 (76%)*
*Pets in Crisis*	*137 (40%)*	*121 (51%)*	*16 (15%)*
*Welfare Boarding*	*37 (11%)*	*31 (13%)*	*6 (6%)*
*seized by RSPCA*	*8 (2%)*	*4 (2%)*	*4 (4%)*
**Inter agency transfer**	**225 (0%)**	**131 (1%)**	**94 (0%)**
**Total**	49,393 (80%) *	22,577 (79%) **	26,816 (83%) ***

* Of all 195,387 admissions, 80% of owner-surrenders had a reason for surrender provided ** Of all adult admissions, 79% had a reason for surrender provided. *** Of all kitten admissions, 83% had a reason for surrender provided.

**Table 7:** Ratios of geometric mean numbers of cat admissions per shelter to 39 RSPCA shelters between June 2006 and July 2010 by calendar month in comparison to December (reference group).

Calendar Month	Ratios of Geometric Mean Numbers Admitted per Shelter		*p*
	Estimated Ratio	(95% CI)	
January	0.93	(0.80 to 1.06)	0.278
February	0.85	(0.74 to 0.98)	0.024
March	0.89	(0.77 to 1.02)	0.094
April	0.83	(0.72 to 0.95)	0.007
May	0.75	(0.65 to 0.86)	<0.001
June	0.61	(0.53 to 0.70)	<0.001
July	0.50	(0.43 to 0.57)	<0.001
August	0.42	(0.36 to 0.48)	<0.001
September	0.45	(0.39 to 0.52)	<0.001
October	0.62	(0.54 to 0.71)	<0.001
November	0.86	(0.75 to 0.99)	0.032
December	Reference group		
